# Estimating Bulk Stomatal Conductance in Grapevine Canopies

**DOI:** 10.3389/fpls.2022.839378

**Published:** 2022-03-18

**Authors:** Mark Gowdy, Philippe Pieri, Bruno Suter, Elisa Marguerit, Agnès Destrac-Irvine, Gregory Gambetta, Cornelis van Leeuwen

**Affiliations:** EGFV, Bordeaux Sciences Agro, INRAE, Université de Bordeaux, ISVV, Bordeaux, France

**Keywords:** bulk boundary layer conductance, net radiation, transpiration, vineyard water-use models, vine water stress, vapor pressure deficit

## Abstract

In response to changes in their environments, grapevines regulate transpiration using various physiological mechanisms that alter conductance of water through the soil-plant-atmosphere continuum. Expressed as *bulk stomatal conductance* at the canopy scale, it varies diurnally in response to changes in vapor pressure deficit and net radiation, and over the season to changes in soil water deficits and hydraulic conductivity of both the soil and plant. To help with future characterization of this dynamic response, a simplified method is presented for determining bulk stomatal conductance based on the crop canopy energy flux model by Shuttleworth and Wallace using measurements of individual vine sap flow, temperature and humidity within the vine canopy, and estimates of net radiation absorbed by the vine canopy. The methodology presented respects the energy flux dynamics of vineyards with open canopies, while avoiding problematic measurements of soil heat flux and boundary layer conductance needed by other methods, which might otherwise interfere with ongoing vineyard management practices. Based on this method and measurements taken on several vines in a non-irrigated vineyard in Bordeaux France, bulk stomatal conductance was estimated on 15-minute intervals from July to mid-September 2020 producing values similar to those presented for vineyards in the literature. Time-series plots of this conductance show significant diurnal variation and seasonal decreases in conductance associated with increased vine water stress as measured by predawn leaf water potential. Global sensitivity analysis using non-parametric regression found transpiration flux and vapor pressure deficit to be the most important input variables to the calculation of bulk stomatal conductance, with absorbed net radiation and bulk boundary layer conductance being much less important. Conversely, bulk stomatal conductance was one of the most important inputs when calculating vine transpiration, emphasizing the usefulness of characterizing its dynamic response for the purpose of estimating vine canopy transpiration in water use models.

## Introduction

Grapevines regulate their water use (i.e., transpiration) in response to changing atmospheric demand and drought stress by regulating the conductance of water through the plant from the soil to the atmosphere (Oren et al., 1999; McElrone et al., 2013, Keller, 2015). This conductance is regulated by various physiologic mechanisms such as control of stomatal aperture and hydraulic conductivity of the vasculature (Lovisolo et al., 2010), with differences in response observed between varieties (Prieto et al., 2010; Costa et al., 2012). This stomata regulation is also affected by changes in plant water status through various physiological mechanisms (Oren et al., 1999; Roelfsema and Hedrich, 2005). Moreover, conductance varies diurnally (Lu et al., 2003; Zhang et al., 2012; Bai et al., 2015) and across the season (Herrera et al., 2021).

Vineyard water use modeling is often conducted using the FAO 56 approach of applying seasonally variable crop coefficients to estimates of evapotranspiration from a hypothetical reference crop (ET_*o*_). Calculation of ET_*o*_ is based on an application of the Penman Monteith (PM) equation, including the assumption of a fixed conductance for the reference crop (Allen et al., 1998). As a result, this approach does not account for changes in conductance in response to changes in atmospheric demand or drought stress. One vineyard water use model adapted from the FAO approach applies a generic adjustment to transpiration as a function of diminishing soil water content (Lebon et al., 2003), but none include a dynamic representation of conductance response to changes in key environmental variables such as net radiation, vapor pressure deficit, or soil water availability. As a first step in developing such representations, a technically robust and implementable methodology for calculating vine canopy conductance in a vineyard setting is needed.

Developed conceptually at the leaf scale, the PM equation calculates latent heat (water vapor) flux from a leaf surface as a function of net radiation absorbed by the leaf, vapor pressure deficit gradients from within the leaf to the atmosphere, and boundary layer and stomatal resistances to this flux (Monteith and Unsworth, 2013). At the field scale, transpiration from a crop canopy can be estimated by conceptually applying the PM equation as if the canopy were a *big leaf* that is horizontally uniform and entirely covers the soil below (Monteith and Unsworth, 2013). At the leaf scale, *stomatal conductance* (*g*_*s*_) is the ratio of water vapor, or carbon flux divided by the concentration gradient driving flux across the boundary layer at the leaf surface (Monteith and Unsworth, 2013) and is often measured by means of a porometer or gas exchange meter on individual leaves. Applying the big leaf approach at the crop canopy scale, *canopy conductance* (*g*_*c*_) can be calculated by rearranging the PM equation and inputting measured canopy transpiration, atmospheric vapor pressure deficits, net radiation absorbed by the canopy, and the within-canopy (bulk) equivalent of boundary layer conductance, with all fluxes and resistances expressed in terms of unit ground area below the canopy (Granier and Loustau, 1994; Granier et al., 2000; Lu et al., 2003). The *g*_*c*_ resulting from this approach is effectively the parallel combination of the *g*_*s*_ of every leaf in the crop canopy (Kelliher et al., 1995).

The big leaf approach using the inverted PM equation has been applied to determining conductance of forest canopies based on sap flow measurement of transpiration from trees (Köstner et al., 1992; Granier et al., 2000; Ewers et al., 2005; Ghimire et al., 2014; Wang et al., 2014; Kučera et al., 2017), and has also been applied to determining *g*_*c*_ of vineyard canopies (Lu et al., 2003; Zhang et al., 2012; Bai et al., 2015). When applying the inverted PM equation in this manner, however, the determination of net radiation absorbed by the canopy must account for heat flux between the canopy and the ground below (Monteith and Unsworth, 2013). Estimating soil heat flux usually involves burying soil heat flux plates, determining soil thermal properties, measurement of temperature and moisture content in the soil profile, and will need to be implemented in multiple locations to account for spatial heterogeneity (Gao et al., 2017). Such instrumentation can be complicated and time consuming to implement properly, particularly in stony soils. The inverted PM equation approach to canopies also requires accounting for aerodynamic boundary layer conductance above the vineyard, involving measurement of wind speed and temperature profiles at multiple heights above the vineyard canopy (Monteith and Unsworth, 2013). Such measurement of soil heat flux and boundary layer conductance can be cumbersome and also interfere with ongoing management practices in a working vineyard. Alternatively, using the big leaf approach and an assumption of strong coupling between the crop canopy and the atmosphere allows for use of a simplified form of the inverted PM equation that does not require input of soil heat flux or aerodynamic boundary layer conductance (Phillips and Oren, 1998; Ewers and Oren, 2000) and has been applied to vineyards (Bai et al., 2015). The assumption of strong coupling between the vineyard canopy and the atmosphere, however, is based on the assumption that temperatures within the canopy (i.e., big leaf) and bulk air temperature above the canopy are similar (Ewers and Oren, 2000; Monteith and Unsworth, 2013), which may not be appropriate depending on meteorological conditions within and above the vineyard canopy.

In vineyards with open canopies, such as with vines cultivated in rows and surrounded by exposed ground, soil evaporation can account for over half of evapotranspiration (Lascano et al., 1992; Heilman et al., 1994). The big leaf model, however, is based on the assumption of uniform spatial distribution of sensible and latent heat flux from the canopy, and if applied to crops with open canopies may lead to anomalous determinations of *g*_*c*_ (Monteith and Unsworth, 2013). In such cases, a modified two-source approach as developed by Shuttleworth and Wallace (1985) can be used to evaluate heat and water vapor flux from the crop canopy separately from the exposed ground surrounding the canopy. This leads to a calculation of *bulk stomatal conductance* (*g*_*bs*_) that does not require the measurement of soil heat flux or boundary layer conductance above the vineyard, greatly simplifying its determination. This *g*_*bs*_ is similar to *g*_*c*_ in that it represents the net effect of the *g*_*s*_ of all leaves in the portion of the vine canopy being considered. This two-source approach has been used to estimate evapotranspiration vineyards that correlated well with eddy covariance measurements (Ortega-Farias et al., 2007, 2010; Ding et al., 2014).

Based on this two-source approach, a methodology is presented here for determination of *g*_*bs*_ for vineyards with open canopies using data from instrumentation having minimal interference with the operations of a working vineyard and avoiding problematic methodologies for measurement of parameters such as soil heat flux and atmospheric boundary layer.

## Methods

The heat and mass transfer theory behind the PM equation and the two-source energy flux model is presented in the literature using resistance to fluxes rather than conductance in order to facilitate the use of Ohms Law analogies (Monteith and Unsworth, 2013). The following presentation also uses resistances (s m^–1^), with results being converted to conductance (m s^–1^) by inversion, with the latter often used in plant physiology literature.

### Heat Flux Model

**FIGURE 1:**
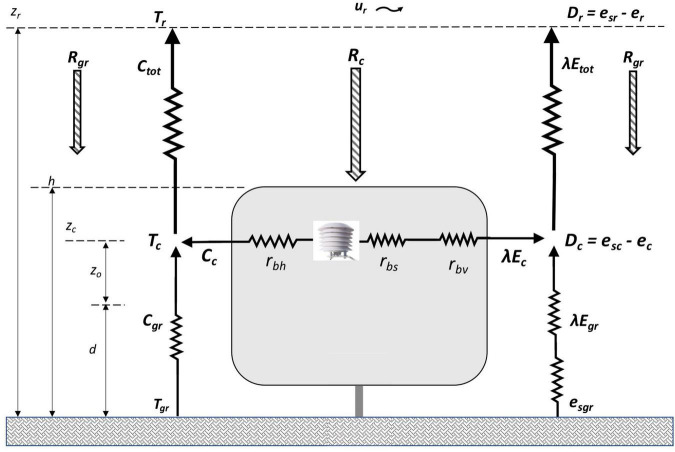
Schematic of convective and radiative heat fluxes to and from the vine canopy and surrounding ground based on Shuttleworth and Wallace, 1985; Lhomme et al., 2012.

For crops with open canopies, Shuttleworth and Wallace (1985) developed a one-dimensional model of sensible and latent heat fluxes separately from the crop canopy and the ground surrounding the canopy as part of a network of temperature and vapor pressure gradients and corresponding flux resistances. Figure 1 presents a simplified schematic of this two-source representation applied to a vineyard with an open canopy.

An important assumption in this method is the *mean canopy height* (*z*_*c*_), or the height at which constituents such as temperature and humidity are considered to be well mixed and uniformly distributed horizontally through the canopy (Shuttleworth and Wallace, 1985). It is the effective height from within the canopy where heat and vapor fluxes can be calculated using a PM type equation (Lhomme et al., 2012). This assumption relies on the open canopies being horizontally consistent across the field and with good aerodynamic mixing within the canopy (Shuttleworth and Wallace, 1985). While *z*_*c*_ is not needed in the calculation of *g*_*bs*_, it was needed to determine the height at which the temperature and humidity sensors in the canopy were placed.

The mean canopy height is determined as the sum of the canopy *zero plane displacement* height (*d*, m) and the *roughness length* (*z*_*o*_, m) (Shuttleworth and Wallace, 1985). Both are related to the apparent drag between the crop canopy and the wind moving over the canopy, with *d* being the height above the ground of the lower asymptote of the wind speed profile above the canopy, and *z*_*o*_ being the height above *d* where the wind speed theoretically goes to zero (Monteith and Unsworth, 2013; Alfieri et al., 2019). Studies have found values of *d* and *z*_*o*_ to be affected by canopy characteristics and differed as a function of wind direction (Chahine et al., 2014), but that flux estimates from a two-source energy balance model were relatively insensitive to such differences (Alfieri et al., 2019). Based on a study in a similarly configured vineyard, values of *d* ranged from 0.62 to 0.75*h* and *z*_*o*_ ranged from 0.08 to 0.14*h*, where *h* is the total height of the canopy above the ground (Chahine et al., 2014). With a vine canopy height at *h* = 1.5 m above the ground, *d* was estimated to be 1m above the ground and *z*_*o*_ at 0.15 m above that, with mean canopy height (*z*_*c*_) being a total of 1.15 m above the ground.

### Bulk Stomatal Conductance

Based on the two-source schematic as shown in Figure 1 an adaptation of the PM equation is used to calculate latent heat flux from the vine canopy (*λE_*c*_*) (Lhomme et al., 2012):


(1) λEc=△⁢Rc+ρ⁢C⁢p⁢(Dc)/rb⁢h△+γ⁢(n+rb⁢srb⁢h)(Wm)-2

where:

*E*_*c*_ = evaporative flux from canopy per unit ground area (g m^–2^ s^–1^)

λ = latent heat of vaporization for water = 2257 (J g^–1^)

*D*_*c*_ = vapor pressure deficit at mean canopy height (Pa)

*R*_*c*_ = net radiation absorbed by the vine canopy per unit ground area (W m^–2^)

*r*_*bs*_ = bulk stomatal resistance (s m^–1^)

*r*_*bh*_ = bulk boundary layer resistance to heat flux (s m^–1^)

*n* = 2 for grapevine leaves with stomata on one side only

γ = psychrometric constant at 1 atm and 20°C = 65.8 (Pa C°^–1^)

Δ = rate of change in saturation vapor pressure versus temperature = 145 (Pa C°^–1^)

ρ*C_*p*_* = heat content per unit volume of air at 20°C = 1212 (Pa C°^–1^)

Equation 1 can then be rearranged to give bulk stomatal resistance:


(2) rb⁢s=△⁢Rc⁢rb⁢h+ρ⁢C⁢p⁢(Dc)λ⁢Ec⁢γ-rb⁢h(△γ-n)(sm)-1

and bulk stomatal conductance is given by inversion:


(3) g=b⁢sr(ms)-1b⁢s-1

The output units for the equations above result when input units shown in the text are used, with all conductance/resistance and fluxes expressed in terms of unit area of vineyard ground attributable to each vine (i.e., row spacing x vine spacing). Multiplying *g*_*bs*_ by the molar volume of air (41.04 mol/m^3^ at 1 atm and 20°C) and converting units gives *g*_*bs*_ in terms of mmol m^–2^ s^–1^, as often used in plant physiology literature.

Conceptually, *r*_*bs*_ is the parallel sum of the leaf level stomatal resistances (*r*_*s*_, s m^–1^) of all individual leaves in the canopy (Kelliher et al., 1995), and calculated as above provides an integrated measure of individual leaf resistances across the range of micro-meteorological conditions experienced by all the leaves in the canopy. A relationship between bulk and leaf-level stomatal conductance is given by the following equation based on leaf area index (*LAI*) and considering whether leaves have stomata on one or both sides (Lhomme et al., 2012):


(4) rb⁢s=n*⁢rs2*⁢L⁢A⁢I(sm)-1

where *n* = 2 for grapevine leaves with stomata on one side only.

If the vine canopy covers more of the ground, however, such as with pergola style trellising or sprawling canopies, use of the big leaf model approach may be more appropriate with measurement of soil heat fluxes from beneath the canopy becoming more important.

### Sensitivity Analysis

A global sensitivity analysis determines the relative importance of the input variables (predictors) to a mathematical model in determining the variation in the output of the model (response), across the range of input values (Iooss and Saltelli, 2017). Such analysis can also be used to characterize the effects of input variable interactions (Razavi and Gupta, 2015; Iooss and Saltelli, 2017). One approach to understanding the relative importance of input variables involves using the output of the model as the response variable in a regression analysis with the inputs to the model as the predictor variables (Saltelli et al., 2008; Razavi and Gupta, 2015).

Such a regression approach is the basis of a global sensitivity analysis of the PM equation using the input data collected for this study. The purpose is to understand the relative importance of the predictor variables in order to prioritize efforts in their determination. For example, the field measurement or estimation of a predictor variable with low importance might be simplified without significantly affecting model results.

A database of *E_*c*_, D_*c*_, R_*c*_*, and *r*_*bh*_ data was first compiled from all vines in the study together with associated *g*_*bs*_ calculated using Eqs 2 and 3. This database was then used for the following two regression analyses:

• *g*_*bs*_ as the response variable with *E_*c*_, D_*c*_, R_*c*_*, and *r*_*bh*_ as predictors, for the purpose of assessing the relative importance of the predictors in a regression model with the same form as the inverted PM equation used to calculate *g*_*bs*_ (Eqs 2 and 3).
• *E*_*c*_ as the response with *g_*bs*_, D_*c*_, R_*c*_*, and *r*_*bh*_ as predictors, for the purpose of assessing the relative importance of the predictors in a regression model with the same form as the regular PM equation used to calculate transpiration (Eq. 1). Of particular interest is the relative importance of *g*_*bs*_ in the determination of *E*_*c*_.

Preliminary review of the data suggested significant multicollinearity in the predictor variables (see correlation matrix in Supplementary Figure S1). Such multicollinearity is not surprising as transpiration (as regulated by changing conductance) is naturally related to vapor pressure deficit and net radiation (Jackson et al., 1981), and solar radiation is related to temperature (Hargreaves and Allen, 2003), and hence vapor pressure deficit.

This multicollinearity, however, can complicate global sensitivity analysis based on classical one-at-a-time, or linear regression methods (Razavi and Gupta, 2015). As an alternative, the random forest non-parametric regression method has been demonstrated as an effective way to perform global sensitivity analysis that is capable of handling interaction between predictors (Grömping, 2009; Antoniadis et al., 2021). A random forest methodology was therefore chosen for the two regression analyses described above.

The random forest methodology processes random selections of predictor variables through a large number of decision trees to find variable relationships that minimize mean square error in the response estimate when compared across many randomly generated test data sets (Breiman, 2001). Unlike parametric (i.e., linear or non-linear) regression methods, non-parametric methods such as random forest do not generate regression coefficients that might otherwise be used to assess the relative importance of predictors. For this purpose, a *minimal depth* approach is used, which quantifies how quickly a predictor variable contributes to determining the response estimate across all the decision trees in the random forest model. The lower the depth number for a predictor, the more important the predictor, with the greatest possible importance having a depth of zero (Ishwaran et al., 2011).

The interactions between predictor variables were also evaluated from the random forest models using a *second order maximal subtree* approach by which *normalized relative minimal depths* of the different predictors are evaluated in a pairwise manner against each other (Ishwaran et al., 2011). The lower the difference in normalized minimal depths between predictors the more closely associated they are, with the interaction effect between variables of high importance having a greater effect on the response variable. For each pair of predictors, this approach generates a normalized index with 0 representing strong interaction and 1 representing none (Ishwaran et al., 2011).

Data analysis was performed using the R software environment (R Project for Statistical Computing, RRID:SCR_001905). Both random forest models were run using the *rfsrc* function of the *randomForestSRC* package for R (Ishwaran and Kogalur, 2021). The hyper parameters for both random forest models were first optimized using the *ranger* function of the *ranger* package for R (Wright et al., 2021). The relative importance of the predictor variables in the determining the response variables in both random forest models are evaluated using the *max.subtree* function of the *randomForestSRC* package for R (Ishwaran and Kogalur, 2021). The interactions between the predictor variables in both random forest models are evaluated using the *find.interactions* function of the *randomForestSRC* package for R (Ishwaran and Kogalur, 2021).

## Materials and Equipment

The measurements for this study were taken on 10 individual grapevines in a vineyard, two each of *Vitis vinifera* L., cv. Cabernet-Sauvignon, Merlot, Tempranillo, Semillon, and Ugni blanc. Measurements of sap flow, temperature and humidity, and solar radiation were taken or interpolated to 15-minute intervals from June 30 to September 15, 2020 and canopy characteristics were measured periodically through the season.

### Vineyard and Canopy Characteristics

The study was performed in a 0.6-hectare common garden experimental vineyard in Bordeaux, France (44° 47′ 0′′ N, 0° 34′ 39′′ W) with 52 varieties planted in a randomized block design. The vines are trained on a vertical shoot positioning trellis system with double Guyot pruning. The top and bottom of the vine canopy are 1.5 and 0.5 m above the ground, respectively, and 0.4 m wide, with canopy dimensions maintained by hedging twice during the growing season. Vine rows are orientated north-south with 1.8 m row spacing and 1.0 m vine spacing. The vines were planted on SO4 rootstock and the soils are clay-gravel typical for the Pessac-Léognan wine appellation (Destrac-Irvine and van Leeuwen, 2016). From 1991 through 2020 average annual total rainfall and reference evapotranspiration were 902 mm and 929 mm, respectively, with annual total solar radiation of 4,790 MJ m^–2^ and average maximum daily temperature from May through June of 25.5°C.

Leaf area was measured at three separate times during the season, in the first halves of July, August and September, respectively. Leaf area was determined by measuring the length and width of all the individual leaves on a subset (approximately 25%) of primary and secondary shoots of each vine. Leaf length and width dimensions were well correlated with individual leaf area as measured by a leaf area meter (Model LI-3100 LICOR Inc., Lincoln, NE, United States) before field measurements began. An average leaf size was calculated from all these individual leaf area measurements segregated based on primary or secondary shoots. These average leaf areas were then applied to a count of all leaves on the remaining primary and secondary shoots on each vine. Leaf area index (*LAI*, m^2^ m^–2^) is calculated as the total leaf area (m^2^) for a vine divided by the area of vineyard ground attributable to each vine (i.e., row spacing × vine spacing). The porosity of each vine canopy was measured in the vineyard using a camera phone application (CANAPEO, Oklahoma State University Department of Plant and Soil Sciences, Stillwater, OK, United States) and interpolated linearly between measurements dates.

### Transpiration Flux (*E*_*c*_)

Heat balance sap flow sensors (Model SGEX, Dynamax Inc., Houston, TX, United States) were installed on one of the two canes of each vine, which were trained to the bottom trellis wire in a double Guyot manner at 50 cm above the ground. Based on manufacturer recommendations, the location of the sap flow sensor on a cane needed to be such that the flow of at least 30% of the vine’s total shoots would be captured by the sensor. It was also found that the quality of the sensor readings benefited from emphasizing installation on straight and smooth cane internodes and by protecting sensors well against the rain.

Sap flow (g s^–1^) was calculated from sensor signals collected by a datalogger (Model SapIP, Dynamax Inc., Houston, TX, United States) and then scaled up for the whole vine based on the ratio of leaf area of the whole vine to the leaf area of shoots downstream the sap flow sensor. Sap flow (g s^–1^) was then divided by the area of vineyard ground attributable to each vine (i.e., row spacing x vine spacing) to give canopy evaporation (transpiration) flux, *E*_*c*_ (g s^–1^ m^–2^).

### Vapor Pressure Deficit (*D*_*c*_)

Saturation vapor pressure, *e*_*sc*_ (Pa) at mean canopy height (*z*_*c*_ = 1.15 m above the ground) was calculated by Teten’s equation using measured temperature *T*_*c*_ (°C) with the partial vapor pressure, *e*_*c*_ (Pa) calculated from *e*_*sc*_ using measured relative humidity. Temperature and humidity were measured using TinyTag Plus 2 probe/data loggers (Model TGP-4505 by Gemini Data Loggers, Chichester, West Sussex, England) with the temperature/relative humidity probes installed inside solar radiation shields (Model RS3 by Prosensor, Amanvillers, France) and hung from a trellis wire in the vine canopy at the mean canopy height.

#### Net Radiation Flux Absorbed by the Vine Canopy (*R*_*c*_)

The radiation flux (energy/time/unit area) incident on a crop canopy comes from sources of both shortwave solar radiation and long wave heat radiation in the environment. The net (intercepted minus reflected) radiation absorbed by the canopy is the sum of net shortwave radiation and net long wave radiation (Allen et al., 1998).

##### Shortwave Radiation

The method of Riou et al. (1989) was used for estimating the amount of such radiation absorbed by grapevine canopy. This radiation model was also used in the water balance model developed by Lebon et al. (2003). This method models the net shortwave radiation absorbed by a vine canopy as the sum of shortwave radiation from: (i) direct radiation from the sun; (ii) diffuse radiation scattered by the atmosphere or clouds; and (iii) both beam and diffuse radiation reflected from the soil in the space between vine rows minus that reflected again by the leaves of the canopy. The model outputs radiation flux (W m^–2^) of shortwave radiation absorbed by the vine canopy expressed in terms of the unit area of vineyard ground attributable to each vine (i.e., row spacing × vine spacing) (Riou et al., 1989).

The method relies on inputs of canopy dimensions, row spacing, and vine spacing and accounts for an assumed, or measured porosity of the canopy. For the direct component of global radiation absorbed by the canopy, solar angles, such as the hour and height angles, were calculated for input to the model based on the latitude and longitude of the study site (Kalogirou, 2014; Widén and Munkhammar, 2019). Based on measurements of global (shortwave) radiation incident on the vineyard, and applying the approach described in Liu and Jordan (1960), the relative amounts of direct and diffuse shortwave radiation were calculated on 15-minute intervals over the season for input to the model. Global (shortwave) radiation flux was measured at a weather station next to the vineyard using a horizontally mounted pyranometer (Model No. CMP6 by Kipp & Zonen, Delft – Netherlands) on one-hour intervals, and then linearly interpolated to 15-minute intervals for the above calculations.

##### Long Wave Radiation

Long wave (heat) radiation from the atmosphere, surrounding ground, and adjacent vine canopy rows are also a source of radiation for the vine canopies (Pieri, 2010). The proportion of radiation flux from the sky, ground, and adjacent vine rows incident on the vine canopy were calculated using radiation view factors between these sources and the faces of the vine canopy. The amount of radiation flux intercepted by the canopy was then adjusted to account for canopy porosity. The canopies themselves were also assumed to radiate heat energy as a function of their temperature. The net long wave radiation absorbed by the vine canopy was then the sum of the amount absorbed from all sources, minus the amount radiated from the canopy, together expressed as long wave radiation flux (W m^–2^) in terms of unit area of vineyard ground attributable to each vine (i.e., row spacing x vine spacing).

Long wave radiation flux from the sky (W m^–2^) was measured on 15-minute intervals with an upward facing pyrgeometer (Model No. SL-510-SS, Apogee Instruments, Logan, UT, United States) on a mast 1.5 m above the vine canopy. Long wave radiation flux from the surrounding ground was determined by measurement of ground temperature using a rectangular field of view infrared radiometer (Model No. SI-1H1 IR, Apogee Instruments, Logan, UT, United States) and converted to long wave radiation flux (W m^–2^) using the Stefan-Boltzmann equation with an assumed emissivity for dry grass of 0.98 (Rubio et al., 1997). The long wave energy radiated by the vine canopy and intercepted from adjacent vine canopies were calculated from the temperatures measured in the canopy and application of the Stefan-Boltzmann equation with an assumed emissivity for vine foliage of 0.98 (Jones et al., 2003; Pieri, 2010).

#### Bulk Boundary Layer Resistances to Heat and Vapor Flux (*r*_*bh*_ and *r*_*bv*_)

Development of Eq. 1 in the literature uses the bulk boundary layer conductance to heat flux (*r_*bh*_*, s m^–1^) as opposed to bulk boundary layer resistance to vapor flux (*r_*bv*_*, s m^–1^) based on the relationships in Eqs 5 and 6 below, in which *n* = 2 for grapevine leaves with stomata on one side only. Similar to bulk stomatal resistance, the bulk boundary layer resistances to heat and water vapor flux are canopy-level summations of the leaf-level boundary layer resistances across all leaves in the canopy stated in terms of unit ground area (Lhomme et al., 2012):


(5) rb⁢h= rb⁢l2*⁢L⁢A⁢I(sm)-1

(6) rb⁢v=n*rb⁢h= 2*⁢rb⁢l2*⁢L⁢A⁢I(sm)-1

where:

*r_*bl*_* = is the one-sided leaf-level boundary layer resistance (s m^–1^).

*LAI* = leaf area index.

One method of estimating *r*_*bl*_ requires the determination of wind speed at the top of the crop canopy (Choudhury and Monteith, 1988; Lhomme et al., 2012). This can be inferred from wind speed profiles measured at multiple heights above the crop canopy in the atmospheric boundary layer (Monteith and Unsworth, 2013). As an alternative, a basic assumption of *r_*bl*_* = 25 s m^–1^ was proposed by Shuttleworth and Wallace (1985) and demonstrated to be adequate based on the relatively low importance of *r*_*bh*_ in the PM equation. The low importance of *r*_*bh*_ in the calculation of *g*_*bs*_ is also confirmed in the sensitivity analysis presented in the next section. This assumption of *r_*bl*_* = 25 s m^–1^ has the additional benefit of avoiding the need for wind speed measurements as needed to determine the wind speed profile above the canopy. In climates with different prevailing temperature, vapor pressure deficit, or wind conditions, however, it may be found that r_*bh*_ is more important than presented here, in which case measurement of wind speeds may be beneficial.

#### Predawn Leaf Water Potential

Predawn leaf water potential (Ψ_*PD*_) measurements provide the water potential of the plant at night when the stomata are closed and the plant is in equilibrium with the root zone water potential (Choné et al., 2001) and is an accepted plant-based measurement of plant water stress (Sperry et al., 1996). Measurements of Ψ_*PD*_ were taken on each vine in the study at six times, roughly 10–14 days apart depending on weather, from early July through early September 2020. Leaf sampling and measurement were done early enough to ensure all was completed no later than 30 minutes prior to sunrise. Measurements were taken by the pressure chamber method of Scholander et al. (1965) using a pressure chamber with digital manometer (DG MECA, 33175 Gradignan, France).

## Results and Discussion

### Bulk Stomatal Conductance

**FIGURE 2:**
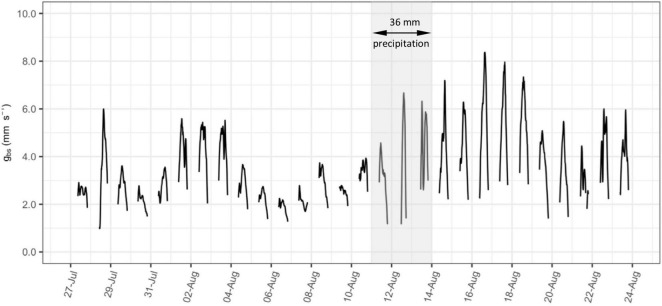
Example timeseries of bulk stomatal conductance (*g*_*bs*_, mm s**^–^**^1^) calculated on 15-minute intervals from a selected vine (C6-2) between 27 July and 24 August, 2020. Shading indicates days over which there was 36 mm of precipitation.

The time series of bulk stomatal conductance (*g*_*bs*_, mm s^–1^) calculated on 15-minute intervals is presented as an example in Figure 2 for vine C6-2 between 27 July and 24 August 2020. Gaps in this 15-minute interval time series are caused by filtering of data when net radiation absorbed by vine canopy (*R*_*c*_) is less than 50 W m^–2^ during the early morning and evening, or at night. The calculation of *g*_*bs*_ using Eqs 2 and 3 is prone to inaccuracy at low levels of *R*_*c*_, and corresponding low levels of *D*_*c*_ and *E*_*c*_. This approach of filtering low solar radiation data was also used in previous studies of conductance in vineyards (Lu et al., 2003; Zhang et al., 2012).

The diffusion rate of water vapor through stomata (i.e., stomatal conductance) is affected by: (i) solar radiation, which adds energy to the diffusion; (ii) vapor pressure deficit, which is the driving force for diffusion; and (iii) the effects of boundary layer resistance to diffusion at the leaf surface (Keller, 2015). At the canopy scale, diurnal fluctuations in *R*_*c*_ and *D*_*c*_ are likely responsible for the strong diurnal variation in 15-minute *g*_*bs*_ also observed in Figure 2, particularly on days with higher overall levels of conductance. The effect of boundary layer resistance, as accounted for in *r*_*bh*_, however, will be largely a function of changes in leaf area over the course of the season.

**FIGURE 3:**
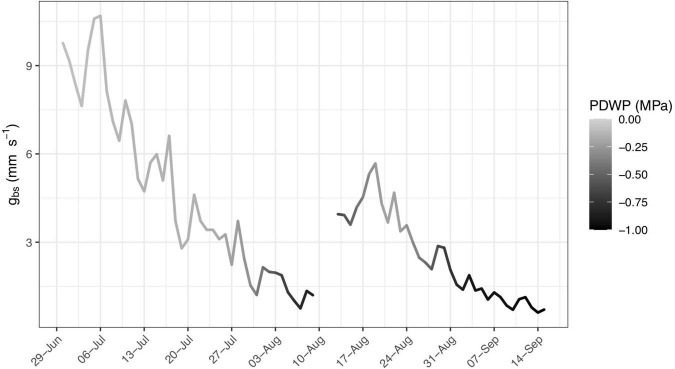
Daily maximum hourly running average of 15-min estimates of the bulk stomatal conductance, *g*_*bs*_ (mm s**^–^**^1^) from a selected vine (C5-2) between 30 June and 15 September, 2020 with shading of line representative of corresponding measured predawn leaf water potential varying from 0.0 MPa (light gray) to –1.0 MPa (black).

Stomata regulation is also affected by changes in plant water status (Oren et al., 1999; Roelfsema and Hedrich, 2005), with predawn leaf water potential (Ψ_*PD*_) providing a useful measure (Sperry et al., 1996; Choné et al., 2001). The effect of Ψ_*PD*_ on *g*_*bs*_ is observed in Figure 3, which presents the daily maximum hourly running average of 15-minute estimates of *g*_*bs*_ from a selected vine (C5-2) between 30 June and 15 September 2020, with shading of the line representing the corresponding measured Ψ_*PD*_, varying from 0.0 MPa (light gray) to −1.0 MPa (black). The lowest levels of *g*_*bs*_ are observed when Ψ_*PD*_ is more negative. The shading associated with corresponding Ψ_*PD*_ measurements is also included in the plots of daily maximum hourly running average of 15-minute estimates of *g*_*bs*_ for all ten vines in Supplementary Figure S2.

Under heterogeneous moisture conditions, predawn water potential measurements have been found to equilibrate at less negative values with portions of the root zone having higher moisture content (Améglio et al., 1999), as may happen in a dry soil after a rainfall. Both Figures 2, 3 show a noticeable increase in overall levels of conductance for a few days after the only significant rainfall of the season (36 mm between 11–13 August). This may be the result of less negative Ψ_*PD*_ resulting from infiltration of rainfall into the upper part of the root zone. A similar increase in conductance after this rainfall was observed in the daily maximum hourly running average of *g*_*bs*_ for most of the ten vines as presented in Supplementary Figure S2.

The maximum observed *g*_*bs*_ over the season for each of the 10 vines in this study ranged from around 5 to 10 mm s^–1^ with occasional peaks from around 10 to 15 mm s^–1^. This compares well with canopy conductance up to 7.0 mm s^–1^ determined on cv. Sultana (Lu et al., 2003), values up to 8.5 mm s^–1^ determined on cv. Merlot (Zhang et al., 2012), and upward of 16 mm s^–1^ in morning hours and 12 mm s^–1^ in the afternoon hours determined on cv. Thompson Seedless (Bai et al., 2015). The differences in maximum observed *g*_*bs*_ between individual vines at a given time may be due to a combination of factors, such as differences in individual vine root access to water and hence vine water status, and genetic differences between varieties in stomatal response to changes in *R*_*c*_ and *D*_*c*_.

A multi variable analysis of the above factors and others is beyond the scope of this paper, although the methodology presented here provides a useful means of determining *g*_*bs*_ for such future analysis.

### Sensitivity Analysis and Variable Interactions

Two optimized random forest regression models, one model with *g*_*bs*_ as the response variable and the other with *E*_*c*_ as the response variable were created using the combined data from all 10 vines in the study. The minimum depths and pairwise variable interactions for the predictors from both of these random forest models are presented in Table 1.

**TABLE 1:** Predictor variable minimum depths and pairwise relative minimum depths between predictor variables for two regression models: **(A)** with *g*_*bs*_ as the response variable; and **(B)** with *E*_*c*_ as the response variable.

**(A) With *g*_*bs*_ as response variable.**					
	**Minimum**	**Pairwise relative minimum depths**			
**Variable**	**Depth**	***E*_*c*_**	***D*_*c*_**	***R*_*c*_**	***r*_*bh*_**

*E*_*c*_	0.00	—	0.05	0.29	0.31
*D*_*c*_	1.00	0.05	—	0.26	0.29
*R*_*c*_	6.20	0.11	0.11	—	0.12
*r*_*bh*_	6.80	0.11	0.11	0.12	—

**(B) With *E*_*c*_ as response variable.**					
	**Minimum**	**Pairwise relative minimum depths**			
**Variable**	**depth**	***g*_*bs*_**	***D*_*c*_**	***R*_*c*_**	***r*_*bh*_**

*g*_*bs*_	0.00	—	0.07	0.14	0.25
*D*_*c*_	1.62	0.05	—	0.18	0.20
*R*_*c*_	3.00	0.07	0.07	—	0.11
*r*_*bh*_	5.37	0.10	0.09	0.11	—

Based on the minimum depth methodology, a predictor with a minimum depth of 0 is the most important in determining the response of the model, with the other predictors being relatively less important as their corresponding minimum depths increase (Ishwaran et al., 2011). For the model with *g*_*bs*_ as the response variable, Table 1(A) indicates that *E*_*c*_ is the most important predictor. Due to this importance, proper implementation of sap flow measurements to determine transpiration, and hence *E*_*c*_ should be emphasized. Conversely, in the model with *E*_*c*_ as the response variable, Table 1(B) indicates *g*_*bs*_ was the most important predictor, confirming the importance of properly characterizing *g*_*bs*_ when modeling the vine canopy component of transpiration in vineyard water balance models.

After *E*_*c*_ and *g_*bs*_, D_*c*_* is a significant predictor in both regressions, suggesting the quality of temperature and humidity measurements in the vine canopy and proper placement of instruments at the mean canopy height (*z*_*c*_) should be emphasized. *R*_*c*_ was of relatively low importance in both regressions, and therefore a simplification in its method of estimation is proposed in the next section. The least important predictor in both regressions was *r*_*bh*_, further justifying use of the simplified assumption of *r*_*bl*_ = 25 s m^–1^ as described earlier according to Shuttleworth and Wallace (1985) in Eqs 5 and 6, and eliminating the need for measurement of wind speed above the canopy.

The normalized pairwise relative minimum depths between predictor variables are also presented in Tables 1(A,B) for both regression models. The normalized depths (with 0 indicating strong interaction and 1 indicating no interaction) are shown for a given predictor variable in each row, with the strength of the interaction being greater for predictor variables of higher importance (Ishwaran and Kogalur, 2021). The interactions between *E*_*c*_ and *D*_*c*_, the most important variables in the regression model for *g*_*bs*_, are very strong at 0.05 in both pairings. Likewise, the interactions between *g*_*bs*_ and *D*_*c*_, the most important variables in the regression model for *E*_*c*_, are also strong at 0.05 and 0.07 for the two pairings. Interactions are observed between other variables, but they are less significant because of the lower importance of those variables. It should be noted, however, the results of the sensitivity analysis could vary with different ranges of *D*_*c*_, *R*_*c*_, wind speed, or *LAI* as might be experienced in different years, locations, or vineyards with different designs or subject to different management practices.

The finding of significant interactions between important variables confirms the preliminary observations in the correlation matrix in Supplementary Figure S1 and supports the use of the random forest regression approach for the sensitivity analysis, which is capable of handling such variable interactions. Such strong interaction may also affect any additional regression analysis aimed at characterizing the relationship between *g*_*bs*_ and other environmental variables.

### Net Radiation (*R*_*c*_)

**FIGURE 4:**
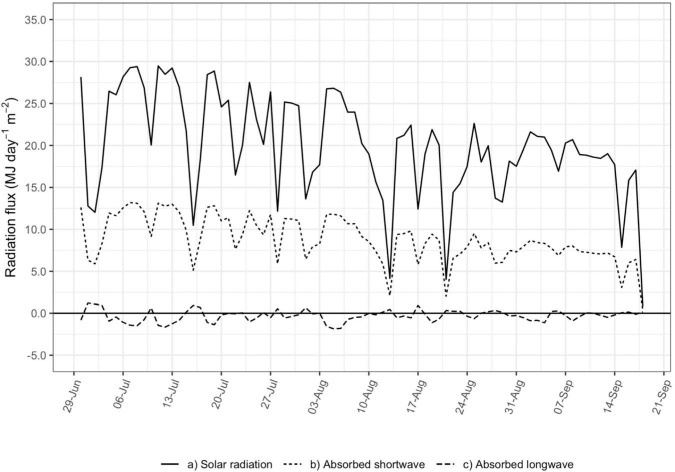
Daily shortwave and long wave radiation flux (MJ day**^–^**^1^ m**^–^**^2^) absorbed by the vine canopy (dashed lines) and solar radiation flux (MJ day**^–^**^1^ m**^–^**^2^) incident on the vineyard (solid line).

Figure 4 presents modeled net shortwave and net long wave radiation flux absorbed by the vine canopy and the measured global radiation flux incident on the whole vineyard, expressed in terms of the amount of energy absorbed by the vine canopy per day per unit vineyard ground area (MJ day^–1^ m^–2^). Daily fluxes were tallied with values of 15-minute *R*_*c*_ greater than 50 W m^–2^ to match the filtering used in the calculation of *g*_*bs*_.

The shortwave radiation absorbed by the canopy follows closely with global radiation incident on the vineyard and represents the majority of the total radiation absorbed by the vine canopy. Net long wave radiation averaged close to zero on most days, although it could represent up to 30% of total net radiation when shortwave radiation is low. This is associated with cloudy conditions when transpiration is already low and not contributing much to vineyard water use.

For that reason, and because of the relatively low importance of *R*_*c*_ in the regressions with either *g*_*bs*_ or *E*_*c*_, net long wave radiation absorbed by the vine canopy was disregarded in the estimation of *R*_*c*_ used in the final calculations of *g*_*bs*_. This has the benefit in the future of eliminating instrumentation in the vineyard for measurement of long wave radiation from the sky and surrounding ground. Similarly, other studies of conductance in vineyards did not account for long wave radiation in determining net radiation (Lu et al., 2003; Zhang et al., 2012; Bai et al., 2015). However, if *R*_*c*_ is found to be a more important variable in the determination of *g*_*bs*_ or *E*_*c*_, then measurement of long wave radiation may be beneficial, perhaps as in cloudier climates.

## Conclusion

Based on the two-source energy flux approach of Shuttleworth and Wallace (1985), a methodology is presented here for determination of *g*_*bs*_ for vineyards with open canopies, using measurements of: (i) vine transpiration measured by sap flow as needed to calculate transpiration flux from the canopy; (ii) measurements of temperature and relative humidity in the vine canopy as needed to calculate vapor pressure deficit; (iii) solar radiation measures as needed to estimate the net radiation absorbed by the vine canopy; and (iv) vine canopy dimensions, porosity, and leaf area as needed for the above. Diurnal variations in 15-minute estimates of *g*_*bs*_ were observed, with maximum daily levels comparing well with previously published values for grapevines the literature. Decreases in *g*_*bs*_ over the season were also observed in association with more negative Ψ_*PD*_.

This methodology respects the energy flux dynamics of vineyards with open canopies, while avoiding involved and error-prone measurements that can also interfere with the operations of a working vineyard. It does not require measurement of soil heat flux, boundary layer conductance above the vineyard, nor long wave radiation, although consideration may need to be given to such measurements in other climates or vineyard configurations. It provides a practical means of quantifying conductance for further study of conductance response to changes in atmospheric demand and drought stress.

A global sensitivity analysis using data from the study found *E*_*c*_ and *D*_*c*_ are the most important variables in the determination of *g*_*bs*_, therefore warranting attention in their field measurement. Conversely, *g*_*bs*_ was found to be the most important predictor of *E*_*c*,_ emphasizing the importance of having better representations of conductance response in vineyard water use models.

## Data Availability Statement

The raw data supporting the conclusions of this article will be made available by the authors, without undue reservation.

## Author Contributions

MG and AD-I coordinated and executed field measurements. PP advised on instrumentation and theoretical basis for analysis. MG compiled the dataset and worked with BS on statistical analysis. All authors were involved in design of the study, helped guide its execution, and participated in the writing and editing of the submitted manuscript.

## Conflict of Interest

The authors declare that the research was conducted in the absence of any commercial or financial relationships that could be construed as a potential conflict of interest.

## Publisher’s Note

All claims expressed in this article are solely those of the authors and do not necessarily represent those of their affiliated organizations, or those of the publisher, the editors and the reviewers. Any product that may be evaluated in this article, or claim that may be made by its manufacturer, is not guaranteed or endorsed by the publisher.

## Abbreviations

Energy fluxes: *C*_*c*_: sensible heat fluxes from the canopy
*C*_*gr*_: at the ground
*C*_*tot*_: total
*λ E_*c*_*: latent heat fluxes from the canopy
*λ E_*gr*_*: at the ground
*λ E_*tot*_*: total
*R*_*c*_: net radiation absorbed by the canopy
*R*_*gr*_: by the ground
Dimensions: *h*: height to top of canopy above the ground
*d*: zero plane displacement
*z*_*o*_: roughness length
*z*_*c*_: mean canopy height
*z*_*r*_: reference height
Atmospheric parameters: *T*_*c*_: temperature at mean canopy height
*T*_*gr*_: at the ground
*T*_*r*_: at reference height
*e*_*sc*_: saturation vapor pressure at *T*_*c*_
*e*_*sgr*_: at *T*_*gr*_
*e*_*sr*_: at *T*_*r*_
*e*_*c*_: measured vapor pressure at *z*_*c*_
*e*_*r*_: at *z*_*r*_
*D*_*c*_: vapor pressure deficit (*e*_*sc*_ – *e*_*c*_) at *z*_*c*_
*D*_*r*_: vapor pressure deficit (*e*_*sr*_ – *e*_*r*_) at *z*_*r*_
*u*_*r*_: wind speed at *z*_*r*_
Bulk canopy resistances: *r*_*bs*_: bulk stomatal resistance
*r*_*bh*_: bulk boundary layer resistance to heat flux
*r*_*bv*_: bulk boundary layer resistance to water vapor flux
Water stress: Ψ _*PD*_: pre-dawn leaf water potential.
