# Supplementary figures and images for: Estimating Bulk Stomatal Conductance in Grapevine Canopies
Source: Front Plant Sci. 2022 Mar 18;13:839378. doi: 10.3389/fpls.2022.839378 (PMC8972124; doi:10.3389/fpls.2022.839378)

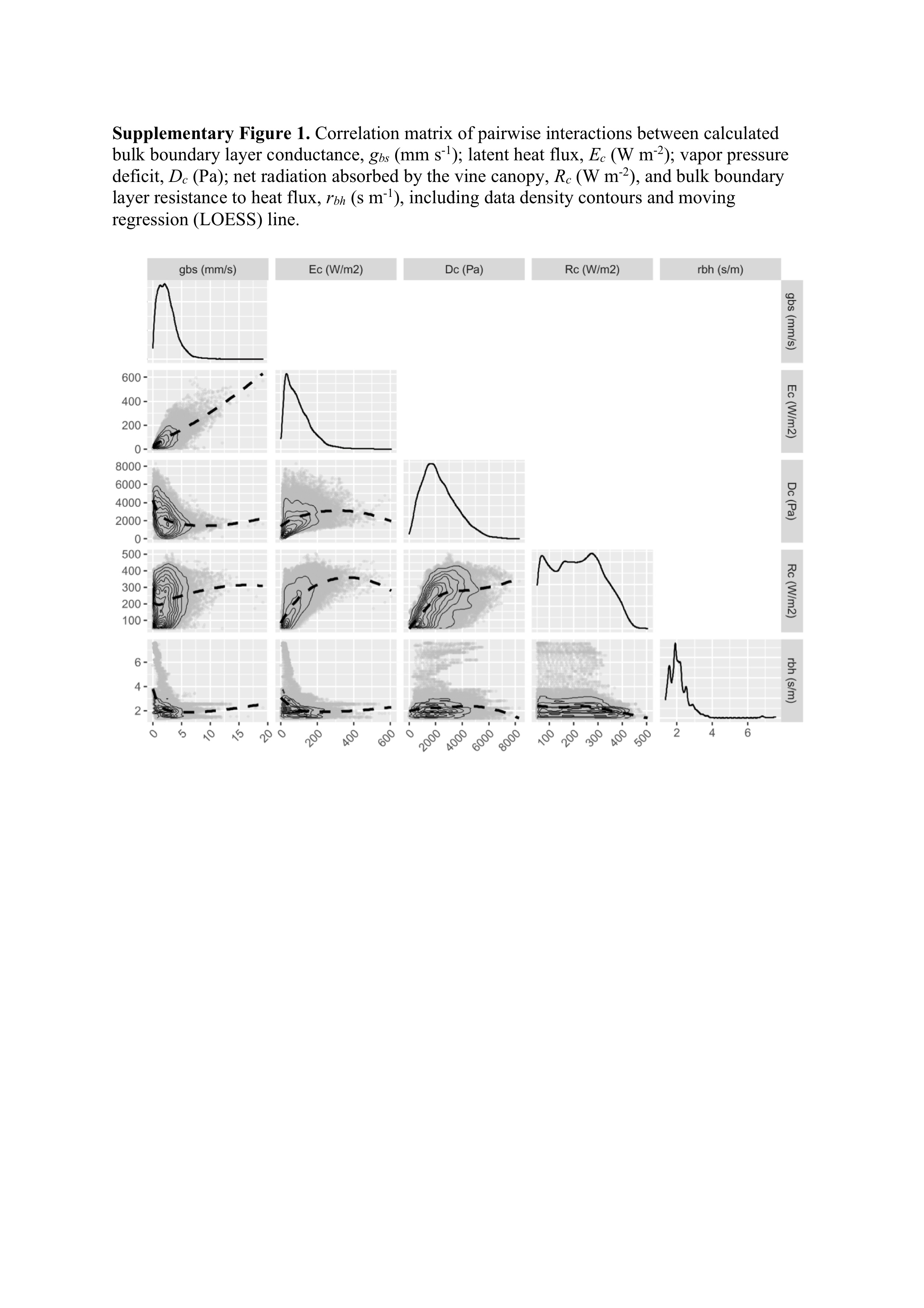

Supplement: Supplementary file 1 [file Image_1.jpg]

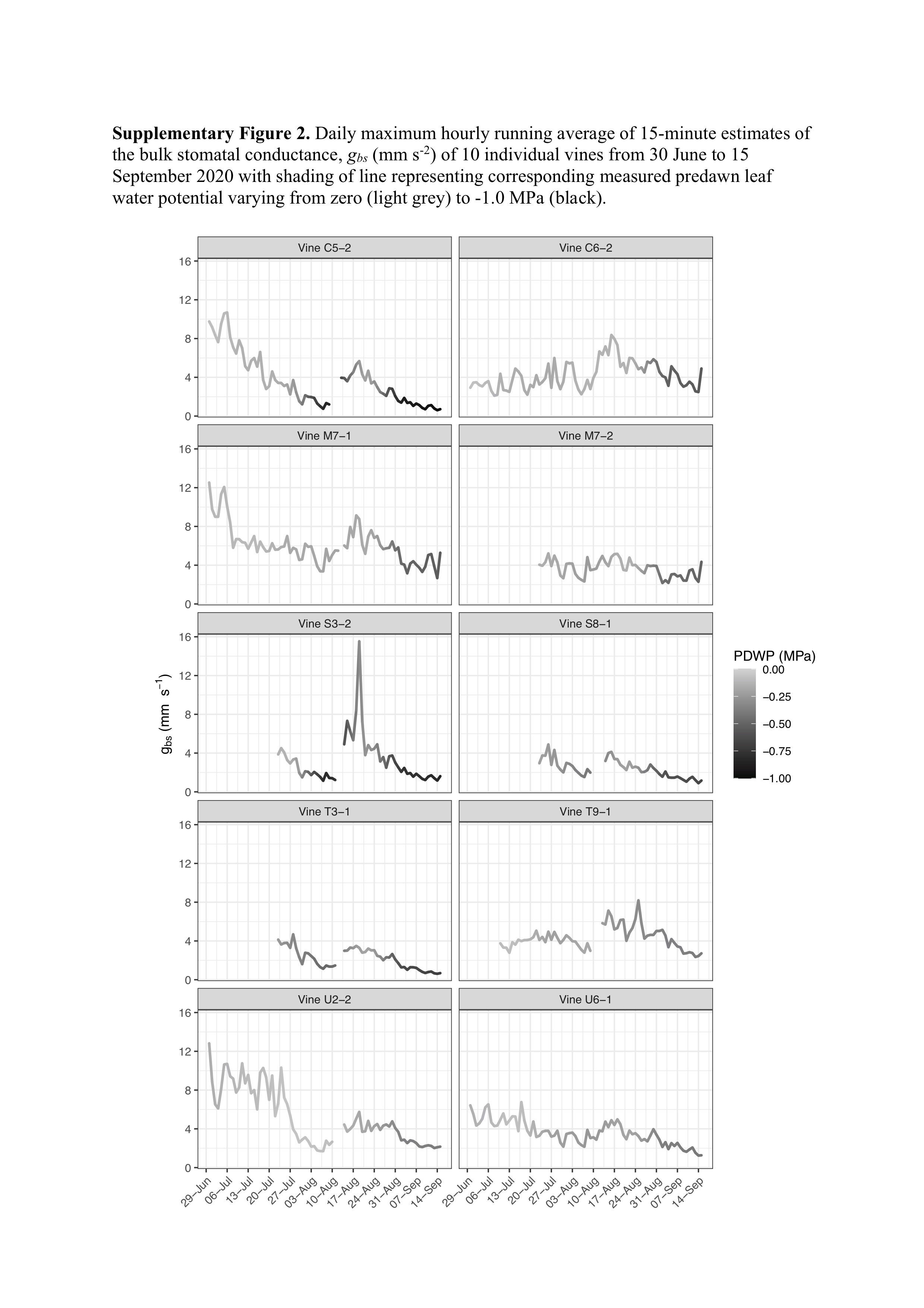

Supplement: Supplementary file 2 [file Image_2.jpg]
